# Effect of CPP-ACP paste with and without CO_2_ laser irradiation on demineralized enamel microhardness and bracket shear bond strength

**DOI:** 10.1590/2177-6709.22.4.053-060.oar

**Published:** 2017

**Authors:** Nasrin Farhadian, Loghman Rezaei-Soufi, Seyed Farzad Jamalian, Maryam Farhadian, Shahrzad Tamasoki, Milad Malekshoar, Bahareh Javanshir

**Affiliations:** 1 Hamadan University of Medical Sciences, Dental Faculty, Orthodontics Department (Hamadan, Iran).; 2 University of Medical Sciences, Dental Research Center, Department of Restorative Dentistry. Hamadan (Hamadan, Iran).; 3 Private practice (Hamadan, Iran).; 4 Hamadan University of Medical Sciences, Department of Biostatistics (Hamadan, Iran).

**Keywords:** Orthodontics, Bonding, Enamel, Casein phosphopeptide-amorphous calcium phosphate, Laser.

## Abstract

**Introduction:**

Many patients seeking orthodontic treatment already have incipient enamel lesions and should be placed under preventive treatments. The aim of this *in vitro* study was to evaluate the effect of CPP-ACP paste and CO_2_ laser irradiation on demineralized enamel microhardness and shear bond strength of orthodontic brackets.

**Methods:**

Eighty caries-free human premolars were subjected to a demineralization challenge using *Streptococcus mutans*. After demineralization, the samples were randomly divided into five equal experimental groups: Group 1 (control), the brackets were bonded without any surface treatment; Group 2, the enamel surfaces were treated with CPP-ACP paste for 4 minutes before bonding; Group 3, the teeth were irradiated with CO_2_ laser beams at a wavelength of 10.6 µm for 20 seconds. The samples in Groups 4 and 5 were treated with CO_2_ laser either before or through CPP-ACP application. SEM photomicrographs of a tooth from each group were taken to observe the enamel surface. The brackets were bonded to the buccal enamel using a conventional method. Shear bond strength of brackets and ARI scores were measured. Vickers microhardness was measured on the non-bonded enamel surface. Data were analyzed with ANOVA and Tukey test at the *p*< 0.05 level.

**Results:**

The mean shear bond strength and microhardness of the laser group were higher than those in the control group and this difference was statistically significant (*p*< 0.05). All groups showed a higher percentage of ARI score 4.

**Conclusion:**

CO_2_ laser at a wavelength of 10.6 µm significantly increased demineralized enamel microhardness and enhanced bonding to demineralized enamel.

## RESUMO

**Introdução:**

muitos pacientes, ao buscar o tratamento ortodôntico, já apresentam lesões incipientes no esmalte e precisam ser submetidos a tratamentos preventivos. O objetivo do presente estudo *in vitro* foi avaliar o efeito da pasta CPP-ACP e da irradiação com *laser* de CO_2_ na microdureza do esmalte desmineralizado e na resistência ao cisalhamento de braquetes ortodônticos.

**Métodos:**

oitenta pré-molares humanos hígidos foram submetidos a desmineralização usando *Streptococcus mutans*. Após a desmineralização, as amostras foram divididas aleatoriamente em cinco grupos experimentais: Grupo 1 (controle), os braquetes foram colados sem qualquer tratamento de superfície; Grupo 2, a superfície do esmalte foi tratada com pasta CPP-ACP por 4 minutos antes da colagem; Grupo 3, os dentes foram irradiados com *laser* de CO_2_ no comprimento de onda de 10,6 µm, por 20 segundos; Grupos 4 e 5, as amostras foram tratadas com *laser* de CO_2_ antes ou durante a aplicação de CPP-ACP. Foram feitas fotomicrografias por Microscopia Eletrônica de Varredura (MEV) de um dente de cada grupo, para avaliação da superfície do esmalte. Os braquetes foram colados ao esmalte na face vestibular, usando-se o método convencional. Foram medidos a resistência ao cisalhamento dos braquetes e o escore do Índice de Adesivo Remanescente (ARI). A microdureza Vickers foi medida nas superfícies do esmalte onde não foi realizada colagem. Os dados foram analisados com ANOVA e teste Tukey ao nível de *p*< 0,05.

**Resultados:**

a média da força de resistência ao cisalhamento e da microdureza do grupo *laser* foi superior à do grupo controle, com diferença estatisticamente significativa (*p* < 0,05). Todos os grupos apresentaram maior porcentagem do escore ARI=4.

**Conclusões:**

o *laser* de CO_2_ no comprimento de onda de 10,6 µm aumentou significativamente a microdureza do esmalte desmineralizado e melhorou a adesão dos braquetes nele.

## INTRODUCTION

Areas of early enamel demineralization are called white spots because of the chalky white color compared to normal enamel.^1^ According to Gorelick et al^2^, 24% of patients referred to orthodontic offices already had white spots before treatment is initiated and half of the patients undergoing fixed orthodontic treatment had non-developmental white spot lesions.^2^ Patients with dmft (number of decayed, missing or filled teeth) >8, plaque index (The number of plaque containing surfaces / The total number of available surfaces ) >3 and early lesions >4 are considered high-risk patients.^3^ It is necessary to control early demineralization areas before starting active treatment in these patients. In addition to mechanical control of oral hygiene, several chemical methods can be used, including different forms of fluoride such as varnishes and fluoride-releasing adhesives and CPP-ACP-containing paste and newer methods such as laser irradiation, which can decrease the risk of demineralization and remineralize previously demineralized enamel.^4^

Casein phosphopeptide (CPP) is a milk-derived protein which keeps high concentrations of calcium and phosphate in white spot lesions. Keeping this supersaturated state of calcium and phosphate is necessary for remineralization of initial lesions.^5^ Reports regarding its effects are contradictory. Some of previous studies have suggested reduced demineralization around orthodontic brackets *in vitro* ^6^ ^,7^ and clinical regression of white spot lesions following topical application of CPP-ACP agents *in vivo*.^8^ On the other hand, a systematic review by Azarpazhooh and Limeback^9^ found little evidence for long-term remineralization effect of CPP-ACP whereas a recent systematic review showed that CPP-ACP was able to remineralize early lesions compared to placebo but its effect was not significant compared to fluoride.^10^

Moreover, different laser types such as CO_2_, Nd:YAG and Er:YAG with different parameters have been used for caries prevention. CO_2_ laser with 9.3, 9.6, 10.3 and 10.6 µm wavelengths have ranked first in caries prevention.^11^ After irradiation with laser, chemical and structural alterations in enamel such as decreased carbonates, fusion and re-crystallization of hydroxyapatite crystals make enamel more resistant to acid attacks.^11^ In addition, it has been shown that laser and topical fluoride have synergistic effects and significantly decrease the rate of enamel decalcification.^12^

Since there are increasing numbers of high-risk patients with multiple white spot lesions seeking orthodontic treatment, whose teeth are exposed to laser beams or CPP-ACP as prophylaxis, the effect of these methods on bracket shear bond strength (SBS) is another dilemma. Reports on the effect of CPP-ACP paste on bonding strength are contradictory but it can possibly interfere with etching. Xiaojun et al^13^ reported higher SBS in the CPP-ACP group whereas Moule et al^14^ reported decreased SBS values after the combined use of carbamide peroxide and CPP-ACP. Exposure to laser beams results in bubble-like depressions on enamel surface like type III etching with acid phosphoric. Some studies have shown that acid-etched surfaces have higher bond strengths than laser-etched surfaces,^15^ while some have shown equal bond strengths.^16^

The aim of this study was to evaluate the effect of CPP-ACP paste with and without CO_2_ laser irradiation on microhardness of demineralized enamel and shear bond strength of orthodontic brackets.

## MATERIAL AND METHODS

A total of 100 human premolars without caries, defects and hypoplastic enamel, extracted for orthodontic reasons, were collected and stored in saline until the start of the study. Any calculus or tissue remnants were cleaned with a scaler. The buccal surface of each tooth was covered with acid-resistant varnish, leaving a 4 × 6-mm window exposed for bracket bonding and microhardness test.

### Demineralization process

Ten tooth samples were immersed in 0.1% thymol solution^1^ in order to avoid contamination with non-experimental bacteria; then they were submitted to demineralization challenge using a caries model.^17^

*Streptococcus mutans* (Clarke, ATCC^®^ 35668^TM^ Manassas, Virginia, USA) was used to grow biofilms on samples and demineralize enamel. Culture medium was prepared by incorporating isolated *Streptococcus mutans* in 5 mL of tryptic soy agar (TSB) with 0.5 McFarland turbidity to 80 mL of sterile brain-heart solution containing 5% sucrose. The teeth were washed twice with 0.9% saline near a flame in a sterile plate and then placed in culture tubes in a jar with 10% partial CO_2_ at 37^o^C. Every 24 h, the teeth were washed twice with sterile saline to remove loosely bound material from the enamel structure. Then they were returned to the new culture medium. This process continued for 10 days.^17^

Microbial-exposed teeth were compared with 10 control non-exposed teeth through surface microhardness test. The samples were mounted in self-cured acrylic resin. Microhardness was assessed with Vickers microhardness testing machine (Micromet 1, Buehler LTD, Lakebluff, Illinois, USA) using a 300-g load with a dwell time of 15 seconds. Three indentations were made for each sample and the mean hardness was recorded as Vickers hardness number.

T-test showed statistically significant differences between demineralized and control teeth (*p*< 0.05, mean difference = 85). Eighty tooth samples were demineralized through this method for 10 days. Then the tooth roots were mounted in self-cured acrylic resin and randomly divided into five equal experimental groups (n = 16) as follows:

» Group 1 (Control): The demineralized enamel surface was not treated with CPP-ACP or CO_2_ laser before bonding of bracket.

» Group 2 (CPP-ACP paste): The demineralized enamel surface was treated with CPP-ACP before bonding. A thin layer of CPP-ACP paste (GC Tooth Mousse, Tokyo, Japan) was applied on enamel surfaces by an applicator and left for 4 minutes. Then it was cleaned with cotton rolls and the remaining paste was allowed to remain for another 3 minutes. Finally it was washed with normal saline. After 6 hours, the topical agent was re-applied to the tooth surface using the same method. This procedure was repeated every day for 5 days; then the samples were immersed in artificial saliva.^4^

» Group 3 (CO_2_ laser): The teeth were irradiated with CO_2_ laser (DEKA Laser Technologies, Florence, Italy) at a wavelength of 10.6 µm, a frequency of 5 HZ, an output power of 0.4 W, an operation time of 0.9 sec. The procedure was carried out by a experienced operator with a uniform scanning motion from an approximate distance of 5 mm from the enamel surface in total time of 20 seconds. After irradiation, the samples were washed with normal saline and immersed in artificial saliva.

» Group 4 (laser before CPP-ACP): The demineralized enamel was first irradiated with CO_2_ laser as explained for Group 3, followed by the same protocol carried out in Group 2.

» Group 5 (laser through CPP-ACP): The demineralized enamel in this group was covered with a thin layer of CPP-ACP paste; after 3 minutes laser beams with the same parameters described for Group 3 were applied through the paste over a period of 20 seconds. Then the samples were washed with saline and immersed in artificial saliva.

**Figure 1:**
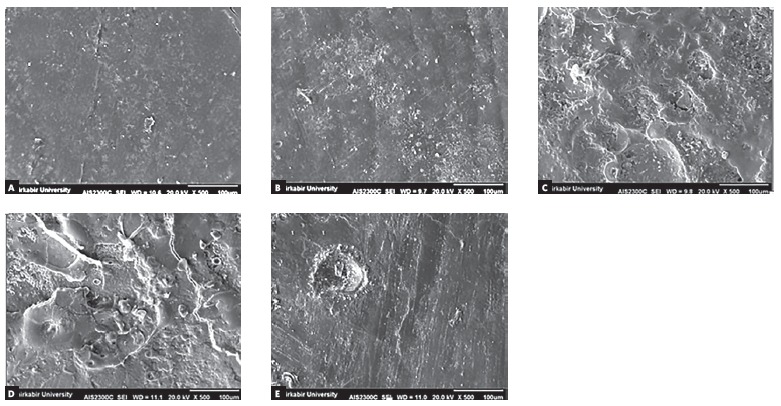
SEM photomicrographs of enamel surface in the five groups. A) Group 1 (control) showed shallow depressions and fine porosities. The enamel surface was devoid of any surface deposits. B) Group 2 (CPP-ACP) demonstrated numerous granular particles; amorphous crystals are arranged on the enamel surface. C) Group 3 (CO_2_ laser) revealed typical melting appearance, cracks and craters with discontinuities. D) Group 4 (laser before CPP-ACP) showed a similar view of laser-irradiated surface with granular and globular particles. E) Group 5 (laser trough CPP-ACP) demonstrated a relatively smooth, more homogeneous surface compared with those of Group 3.

SEM photomicrographs of a representative tooth from each group were taken to observe the enamel surface (Fig 1).

### Bonding of brackets

The exposed enamel of each tooth was etched with 37% orthophosphoric acid (Resilience, Orthotechnology, USA) for 15 seconds, rinsed with water for 15 seconds, and dried with oil-free air for 10 seconds until a frosty white appearance was obtained. Stainless-steel premolar brackets (Dentarum, Germany) were bonded to the teeth using the Transbond XT composite resin (3M Unitek, Monrovia, Calif, USA) according to the manufacturer’s instructions. The bracket was positioned so that the occlusal edge of the bracket was tangent with the occlusal edge of the exposed enamel. An LED light-curing unit (Kerr, DEMI plus, USA) was used for 40 seconds to light-cure the composite resin^4^. Finally the samples were immersed in artificial saliva for 30 days before tests.^4^

### Assessment of bracket shear bond strength 

Shear bond strengths of the samples were tested with a universal testing machine (Santam, STM-20, Iran) using a chisel-edged plunger at a crosshead speed of 1 mm/min. The maximum load that debonded the bracket was recorded in newtons (N) and the SBS was calculated by dividing the force values by the bracket base area (1 MPa=1 N/mm^2^).^18^

Adhesive remnant index (ARI) was assessed and ranked by one investigator as follows:

1 = all the adhesive remaining on the enamel surface;
2 = more than 50% of the adhesive remaining on the tooth surface;
3 = more than 50% of the adhesive remaining on the bracket base;
4 = all the adhesive remaining on the bracket base.^4^

### Surface microhardness test

After completion of SBS assessment, the samples were rinsed with saline solution and mounted in self-cured acrylic resin with the buccal surface parallel to the horizon. A Vickers microhardness tester (Micromet 1, Buehler LTD, Lakebluff, Illinois, USA) was used under a 300-g load and a dwell time of 15 seconds to assess Vickers microhardness. The indenter was placed on a 2 × 4-mm non-bonded exposed enamel surface. For each sample three indentations were made on three points of enamel surface and the mean value was recorded as Vickers hardness number (VHN).

### Statistical analysis

Normality of distribution and homogeneity of variance of values were checked by means of Kolmogorov-Smirnov test. One-way analysis of variance was employed. Tukey *post-hoc* test was carried out to perform multiple comparisons. SPSS software v. 21 (SPSS Inc., Chicago, IL, USA) was used to perform statistical calculations, adopting a significance level of 0.05.

## RESULTS

### Shear bond strength

**Figure 2:**
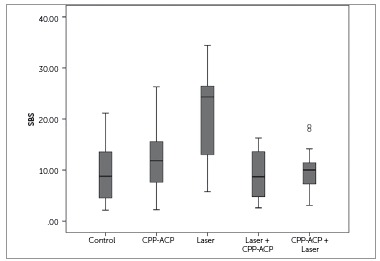
Differences in shear bond strength (SBS) values among the groups.

Kolmogorov-Smirnov test showed that shear bond strength of the teeth in the five groups had a normal distribution. Figure 2 shows the means and 95% confidence intervals in the five groups. Statistical comparisons of the groups are presented in Table 1.

**Table 1:** Multiple comparisons of groups by *post-hoc* Tukey tests for shear bond strength. The mean difference is significant at the 0.05 level

(I) group	(J) group	Mean difference (I-J)	Std. error	Sig.	95% Confidence Interval	
					Lower bound	Upper bound
control	CPP	-3.00533	2.32396	.696	-9.5128	3.5021
	Laser	-11.09867*	2.32396	.000	-17.6061	-4.5912
	Laser + CPP	.48000	2.32396	1.000	-6.0274	6.9874
	CPP+ Laser	-.43467	2.32396	1.000	-6.9421	6.0728
CPP	Laser	-8.09333*	2.32396	.007	-14.6008	-1.5859
	Laser + CPP	3.48533	2.32396	.566	-3.0221	9.9928
	CPP + Laser	2.57067	2.32396	.803	-3.9368	9.0781
Laser	Laser + CPP	11.57867*	2.32396	.000	5.0712	18.0861
	CPP + laser	10.66400*	2.32396	.000	4.1566	17.1714
Laser + CPP	CPP + laser	-.91467	2.32396	.995	-7.4221	5.5928

The results of ANOVA indicated statistically significant differences among the five groups (*p*< 0.001).

Tukey HSD test showed that only the mean SBS of Group 3 was significantly higher than the other groups (*p*< 0.001) (Table 1).

### ARI index

All the groups showed a higher percentage of ARI scores 4 and fracture had occurred at enamel-composite interface.

### Surface microhardness

**Figure 3:**
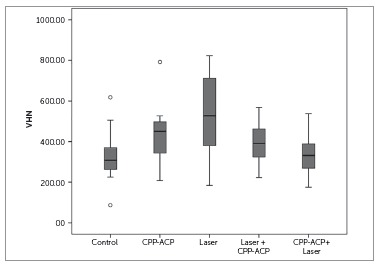
Differences in Vickers hardness (VHN) values among the groups.

Kolmogorov-Smirnov test showed that surface microhardness of teeth in the five groups had a normal distribution. Figure 3 shows means and 95% confidence intervals in the five groups. Statistical comparisons of the groups are presented in Table 2.

**Table 2:** Multiple comparisons of groups by *post-hoc* Tukey tests for Vickers hardness. The mean difference is significant at the 0.05 level.

(I) group	(J) group	Mean difference (I-J)	Std. error	Sig.	95% Confidence Interval	
					Lower bound	Upper bound
control	CPP	-106.79333	51.30682	.240	-250.4603	36.8736
	Laser	-217.19333*	51.30682	.001	-360.8603	-73.5264
	Laser+CPP	-66.86000	51.30682	.690	-210.5270	76.8070
	CPP+Laser	-6.39333	51.30682	1.000	-150.0603	137.2736
CPP	Laser	-110.40000	51.30682	.210	-254.0670	33.2670
	Laser+CPP	39.93333	51.30682	.936	-103.7336	183.6003
	CPP +Laser	100.40000	51.30682	.298	-43.2670	244.0670
Laser	Laser+CPP	150.33333*	51.30682	.036	6.6664	294.0003
	CPP +laser	210.80000*	51.30682	.001	67.1330	354.4670
Laser+ CPP	CPP +laser	60.46667	51.30682	.764	-83.2003	204.1336

The results of ANOVA indicated statistically significant differences among the five groups (*p*< 0.001). Tukey *post-hoc* test showed significantly higher enamel surface microhardness in Group 3 compared with the control group (*p*= 0.001) and Groups 4 and 5 (*p*= 0.036, *p*= 0.001).

## DISCUSSION

The present study assessed the effects of CPP-ACP paste with and without CO_2_ laser irradiation on microhardness of demineralized enamel and bracket shear bond strength at the same time. A microbiological caries model was prepared in this study using *Streptococcus mutans*. Chemical models focus on physiochemical aspects of dental caries but microbiological caries models seem to be more suitable because of more clinically relevant biofilm accumulation around orthodontic brackets.^17^

The hardness of human tooth has been determined by a variety of methods, including abrasion, scratch and indentation techniques.^19^ Vickers microhardness test was used to evaluate remineralization as an indirect test that can measure the changes in surface structural strength from demineralization and remineralization.^19^

There are various *in vitro* studies on prevention and treatment of demineralization with CPP-ACP, but reports are contradictory in terms of designs, time and instructions for use of CPP-ACP. Sudjalim et al,^7^ Uysal et al^4^ and Tantbirojn et al^20^ reported significant prevention and remineralizing effects of CPP-ACP in their *in vitro* studies; on the other hand, Behnan et al,^1^ Heravi et al^21^ and Ballard et al^22^ did not find evidence for positive effect of CPP-ACP on enamel. Use of different study designs, substrates (human or bovine teeth), times of application of CPP-ACP, duration of study and methods of assessing remineralization of enamel - such as microhardness, QLF and photography - can possibly play a role in gaining different results.

A systematic review by Li et al,^10^ in 2014, in which randomized and quasi-randomized clinical trials were included with follow-up periods of 3-24 months with different forms of application of CPP-ACP, showed that only eight articles met the inclusion criteria that evaluated the remineralizing effect of CPP-ACP with direct visualization or bitewing radiographs or photographs. They concluded that CPP-ACP has significant remineralizing effects compared to placebo, but its effect is not significant compared to fluoride. According to these reviews, it seems the effects of CPP-ACP products depend on their type, frequency and duration of application, which are not clearly defined yet.

The laser parameters in the present study were selected according to the study by Esteves-Oliveira et al,^23^ who showed caries inhibition of 81% without destruction of the enamel structure. The present findings were consistent with the results reported by Poosti et al,^12^ Souza-e-Silva^17^ and Miresmaeili et al^11^ that showed, after irradiation with laser, chemical and structural alterations in enamel - such as decreased carbonates, fusion and re-crystallization of hydroxyapatite crystals - make enamel more resistant to acid attacks.

In the present study, the combined effects of CO_2_ laser and CPP-ACP paste on enamel microhardness were also evaluated to assess any synergistic effect considering the mechanism of action of CPP-ACP and also to see if CPP-ACP could neutralize the adverse effects of laser. According to the present results, it appears CPP-ACP has prevented the temperature rise due to laser irradiation and since CPP-ACP alone did not increase the microhardness significantly it is logical that it did not exhibit synergistic effects with laser. To the best of our knowledge, there is only one study^24^ that showed synergistic effects of CO_2_ laser and CPP-ACP on inhibition of demineralization, by polarized light microscopy and profilometry. The study was not carried out on demineralized enamel and also different laser parameters were used. Heravi et al^21^ did not find synergistic effects of CPP-ACP paste and Er:YAG laser and low-level laser on remineralization. On the other hand, Asl-Aminabadi et al^25^ reported synergistic remineralizing effect of CPP-ACP paste and Nd:YAG laser on enamel. Subramaniam et al^26^ also reported increased surface microhardness of teeth after laser irradiation, followed by CPP-ACP application. In both of these studies, primary teeth were used, whose behavior is different in caries and erosion and bonding strength tests.^27^

Because of the increasing number of high-risk patients who seek orthodontic treatment, and their teeth have been exposed to preventive agents because of multiple white spots presence, evaluation of the effect of these methods on bracket bond strength is critical. It is known that teeth with high fluoride content are more resistant to etching.^28^ Reports on the effect of CPP-ACP and CO_2_ laser on bonding strength are contradictory.^14^ ^-^ ^16^ ^,29^ Both can make enamel more resistant to acid and laser causes re-crystallization and melting in enamel structure; therefore, they can possibly interfere with the etching process and affect bond strength.^13^ According to our results, it can be concluded that CPP-ACP has resulted in an enamel surface morphology similar to the controls, which is consistent with the study by Usal et al.^4^ Kecik et al^30^and Xiajoun et al^13^ reported higher bond strengths of CPP-ACP than the controls. This difference can be attributed to higher concentrations and extended time of application of CPP-ACP in these studies and also use of CPP-ACP for prevention on normal enamel. Kecik et al^30^ used bovine enamel that has larger crystal grains and lattice defects due to more rapid development during tooth formation. This may contribute to a lower critical surface tension in bovine enamel.

To the best of our knowledge, there are no reports on the effects of CO_2_ laser pretreatment on brackets’ bond strength to demineralized enamel. Obata et al^31^ proposed CO_2_ laser for debonding instead of etching. Özer et al^32^ reported similar shear bond strength of Er,Cr:YSGG laser-treated surface and acid-etched surface. Talbot et al^33^ reported that argon laser had no effect on shear bond strength when used on enamel before bonding, whereas Farhadian et al^34^ reported that use of argon laser after or through bonding can decrease bracket bond strength. Results of the present study are consistent with the reported etching effect of laser.

Since CPP-ACP alone had no significant effect on the shear bond strength compared to the control group, it seems logical that its combined application had no significant effect and even prevented the laser from affecting shear bond strength.

In the present study, no significant difference was found in ARI scores between the groups and fracture in the adhesive-enamel interface was the most common mode of fracture in all the groups. This can be attributed to the low quality of bonding to demineralized enamel.^4^

According to Reynolds,^35^ SBS values of 5.9 to 7.8 MPa are adequate for orthodontic purposes. In this study, SBS values were higher than this. We tried to use the standard testing procedure to create a laboratory technique which is similar to the clinical situation. However, it is acknowledged that *in vitro* bond strength testing is not a true representative of the intraoral conditions and only guides us to possible clinical effects of the methods that are tested. However, the results assist us in determining which products should be taken to the next level of research.

## CONCLUSION

CO_2_ laser irradiation at a wavelength of 10.6 µm increased demineralized enamel microhardness and also enhanced bonding to demineralized enamel significantly. However, 5-day application of CPP-ACP paste either alone or in conjunction with CO_2_ laser did not increase enamel microhardness significantly compared to the control. All the combinations tested exhibited clinically acceptable bond strengths. Fracture mode of bracket in all the groups was at composite-enamel interface, which might be due to the poor bond between composite resin and demineralized enamel. Further *in-vivo* evaluations are recommended to verify these findings.
